# Funding and Service Organization to Achieve Universal Health Coverage for Medicines: An Economic Evaluation of the Best Investment and Service Organization for the Brazilian Scenario.

**DOI:** 10.3389/fphar.2020.00370

**Published:** 2020-04-14

**Authors:** Marina Morgado Garcia, Pamela Santos Azevedo, Andrew Mirelman, Leandro Pinheiro Safatle, Roberto Iunes, Marion Clark Bennie, Brian Godman, Augusto Afonso Guerra Junior

**Affiliations:** ^1^ Department of Social Pharmacy, Federal University of Minas Gerais, Belo Horizonte, Brazil; ^2^ Collaborating Centre for Health Technology Assessment and Excellence (CCATES), Belo Horizonte, Brazil; ^3^ Centre for Health Economics, University of York, York, United Kingdom; ^4^ Department of Medicines Market Regulation - Brazilian Health Regulatory Agency (ANVISA), Brasília, Brazil; ^5^ World Bank Group, Washington, DC, United States; ^6^ Department of Pharmacoepidemiology, University of Strathclyde, Glasgow, United Kingdom; ^7^ Management School, University of Liverpool, Liverpool, United Kingdom; ^8^ Division of Clinical Pharmacology, Department of Laboratory Medicine, Karolinska Institute, Stockholm, Sweden; ^9^ School of Pharmacy, Sefako Makgatho Health Sciences University, Pretoria, South Africa

**Keywords:** pharmacoecomomics, medicines policy, access to medicines, health inequalities, Universal Health Coverage (UHC), pharmacy funding models, Brazil

## Abstract

**Background:**

There are many health benefits since 31 years after the foundation of the National Health Service (NHS) in Brazil, especially the increase in life expectancy. However, family-income inequalities, insufficient funding, and suboptimal private sector–public sector collaboration are still areas for improvement. The efforts of Brazil to achieve universal health coverage (UHC) for medicines have resulted in increased public financing of medicines and their availability, reducing avoidable hospitalization and mortality. However, lack of access to medicines still remains. Due to historical reasons, pharmaceutical service organization in developing countries may have important differences from high-income countries. In some cases, developing countries finance and promote medicine access by using the public infrastructure of health care/medical units as dispensing sites and cover all costs of medicines dispensed. In contrast, many high-income countries use private community pharmacies and cover the costs of medicines dispensed plus a fee, which includes all logistic costs. In this study, we will undertake an economic evaluation to understand the funding needs of the Brazilian NHS to reduce inequalities in access to medicines through adopting a pharmaceutical service organization similar to that seen in many high-income countries with hiring/accrediting private pharmacies.

**Methods:**

We performed an economic evaluation of a model to provide access to medicines within public funds based on a decision tree model with two alternative scenarios public pharmacies (NHS, state-owned facilities) *versus* private pharmacies (NHS, agreements). The analysis assumed the perspective of the NHS. We identified the types of resources consumed, the amount, and costs in both scenarios. We also performed a budget impact forecast to estimate the incremental funding required to reduce inequalities in access to essential medicines in Brazil.

**Findings:**

The model without rebates for medicines estimated an incremental cost of US$3.1 billion in purchasing power parity (PPP) but with an increase in the average availability of medicines from 65% to 90% for citizens across the country irrespective of family income. This amount places the NHS in a very good position to negotiate extensive rebates without the need for external reference pricing for government purchases. Forecast scenarios above 35% rebates place the alternative of hiring private pharmacies as dominant. Higher rebate rates are feasible and may lead to savings of more than US$1.3 billion per year (30%). The impact of incremental funding is related to medicine access improvement of 25% in the second year when paying by dispensing fee. The estimate of the incremental budget in five years would be US$4.8 billion PPP. We have yet to explore the potential reduction in hospital and outpatient costs, as well as in lawsuits, with increased availability with the yearly expenses for these at US$9 billion and US$1.4 billion PPP respectively in 2017.

**Interpretation:**

The results of the economic evaluation demonstrate potential savings for the NHS and society. Achieving UHC for medicines reduces household expenses with health costs, health litigation, outpatient care, hospitalization, and mortality. An optimal private sector–public sector collaboration model with private community pharmacy accreditation is economically dominant with a feasible medicine price negotiation. The results show the potential to improve access to medicines by 25% for all income classes. This is most beneficial to the poorest families, whose medicines account for 76% of their total health expenses, with potential savings of lives and public resources.

## Introduction

Promoting access to cost-effective, safe, and quality medicines is a priority of public health policies (WHO, 2004). Medicines are consumer goods as well as essential products for health with a key position in economies and in health services. Medicine costs can have a strong and progressive burden on families as a consequence of the increasing costs of available treatments including, for example, cancer medicines in the United States and other essential medicines in developing countries where the cost of medicines can account for 60% or more of total healthcare expenditures (Cameron et al., 2009; Tefferi et al., 2015). Longer life expectancy as a result of new medicines, a higher prevalence of chronic diseases, and typically higher prices for new medicines especially those for cancer and orphan diseases, all contribute to increases in pharmaceutical expenditures in recent years (Howard et al., 2015; Luiza et al., 2016; OECD, 2017; Godman et al., 2018; Luzzatto et al., 2018). The higher prevalence of particularly chronic diseases puts pressure on public budgets to enhance access to medicines, including all essential medicines, exposing the weaknesses of how developing countries, like Brazil, fund and organize their services to distribute prescribed medicines to their citizens (MSH, 2012).

Many high-income countries have promoted health care as a right as part of their efforts to achieve universal health coverage (UHC) for medicines (WHO, 2004). The strategies adopted by high-income countries to fund and organize pharmaceutical services can differ from developing countries. Having said this, there is no universal access to healthcare for all in the US *versus* for instance Western European countries despite the US spending substantially more on health care as a percentage of gross domestic product (GDP) (OECD, 2017). Despite efforts across countries, access to medicines presents challenges for the public supply system in all countries, especially in developing countries. Medicine losses and shortages occur simultaneously with concerns with the quality of pharmaceuticals in several developing countries (Hassali et al., 2014; Fadare et al., 2016; Bochenek et al., 2017; Acosta et al., 2019), representing critical inefficiencies of public services resulting in increased patient expenditures in many countries as well as litigation in some countries where health is a constitutional right (PAHO, 2009; Castro et al., 2019; Nascimento et al., 2017).

Between 2001 and 2014, data from the World Bank showed a substantial increase in public and private health expenditures in many countries. In the Brazilian scenario, this data showed a five times increase in health expenditures (The World Bank, 2019). However, despite this increased funding, there are still concerns with the availability of essential medicines in the public healthcare system with no more than 62% availability of medicines in 2014 (Nascimento et al., 2017), although up from 47% in 2001 (Guerra Júnior et al., 2004). In addition, the lack of access to medicines in ambulatory care may increase treatment expenses for the national health systems as a result of unnecessary progression of diseases which may result in hospitalizations.

Due to historical reasons, pharmaceutical service organization in developing countries can have important differences from high-income countries. In some cases, developing countries finance and promote medicines access by using the public infrastructure of health care/medical units as dispensing sites and cover the costs for each component up to the dispensing of the medicine to patients. This is seen for instance in Malaysia and sub-Saharan Africa where care for patients in the public healthcare system is provided *via* primary healthcare centers or outpatient clinics which can cover the cost of medicines supplied (Rezal et al., 2015; Meyer et al., 2017; Nashilongo et al., 2017; Mbui et al., 2017; Mashalla et al., 2017). In contrast, high-income countries typically use private community pharmacies and cover the costs of the medicines dispensed plus a fee, which includes all logistic costs. In the public services model in Brazil, medicines are provided according to the level of care: basic component—medicines and supplies related to diseases treated in primary health care and specialized component—medicines and supplies for more complex diseases and patients treated by specialists in public and private outpatient services. In the case of specialty medicines, this lack of access can be particularly severe in developing countries as seen by the lack of biological medicines to treat patients with cancer and immunological diseases in Brazil, Central and Eastern European countries, and sub-Saharan African countries (Putrik et al., 2014; Jakupi et al., 2018; Atieno et al., 2018; da Silva et al., 2018; Baumgart et al., 2019; Wilking et al., 2019). Some high-income countries are also now facing difficulties with financing new medicine with continuously increasing prices despite limited incremental benefits for many new medicines (Malmstrom et al., 2013; Howard et al., 2015; Cohen, 2017). One issue that makes the model adopted by the NHS Brazil different from a number of other countries with UHC is that there is no limit on medicine expenditure by families, *i.e.* safety net. This is similar to for instance, Italy and Scotland where there is no copayment for any medicine prescribed and Sweden where there are no patient copayments after an initial limited amount (Godman et al., 2009; Garattini et al., 2016; Leporowski et al., 2018). While the Brazilian Institute for Geography and Statistics’ (IBGE) real-world data shows annually the impact of medicine expenses among all income classes, the current theoretical and legal concept, based on constitutional provision, is that the National Health Service (NHS) could not impose a copayment for medicines or services (IBGE, 2010; IBGE, 2018). The adoption or not of copayment by patients in Brazil were addressed with the implementation of the NHS Federal program in 2003. The program hires private pharmacies and provides medicines with copayments but offers a very short list of medicines, targeting mostly cardiovascular diseases and diabetes.

Overall, the universal healthcare responsibility within countries including Brazil implies the obligation to provide health care using public resources as the primary source of funding. The methods of provision and payment used to achieve UHC may range from pure state services, amalgamation with public and private entities, as well as direct contracting of private providers or social organizations. Regardless of the type of organization and provision adopted, public or private contracted, it will be necessary to establish a system of payments. Whether the provider is public or private, the funding system should have the metrics to remunerate the service provider (pharmacies) to cover their costs. Other factors are the need to balance economic incentives and avoid cost escalation while at the same time promoting equitable access, delivering quality care and products, and ensuring citizens’ satisfaction. The conceptual models for pharmaceutical payment systems include:

- Payment for inputs/resources: all resources consumed to produce pharmaceutical services (HR, infrastructure, logistics, medicines, *etc.*);
- Process Payment: types of procedures and health care performed;
- Payment for Production: quantity and types of medicines supplied to patients;
- Payment by affiliated population: number of people registered in the health region of the provider;
- Payment for Health Results: number of healthy or satisfied patients.

As mentioned, high-income countries typically follow the pay-per-production model where payments and pharmaceutical logistics are fully outsourced. The copayment and safety-net concepts in which households pay up to a preset threshold for medicines over a period are adopted in many countries including Australia, Canada, Denmark, England, Germany, Norway, Portugal, Spain, and Sweden (Pontén et al., 2017; WHO, 2019; Glover, 2017), with, as mentioned, some countries adhering to full, free access to reimbursed medicines (Garattini et al., 2016; Leporowski et al., 2018).

**Figure 1:**
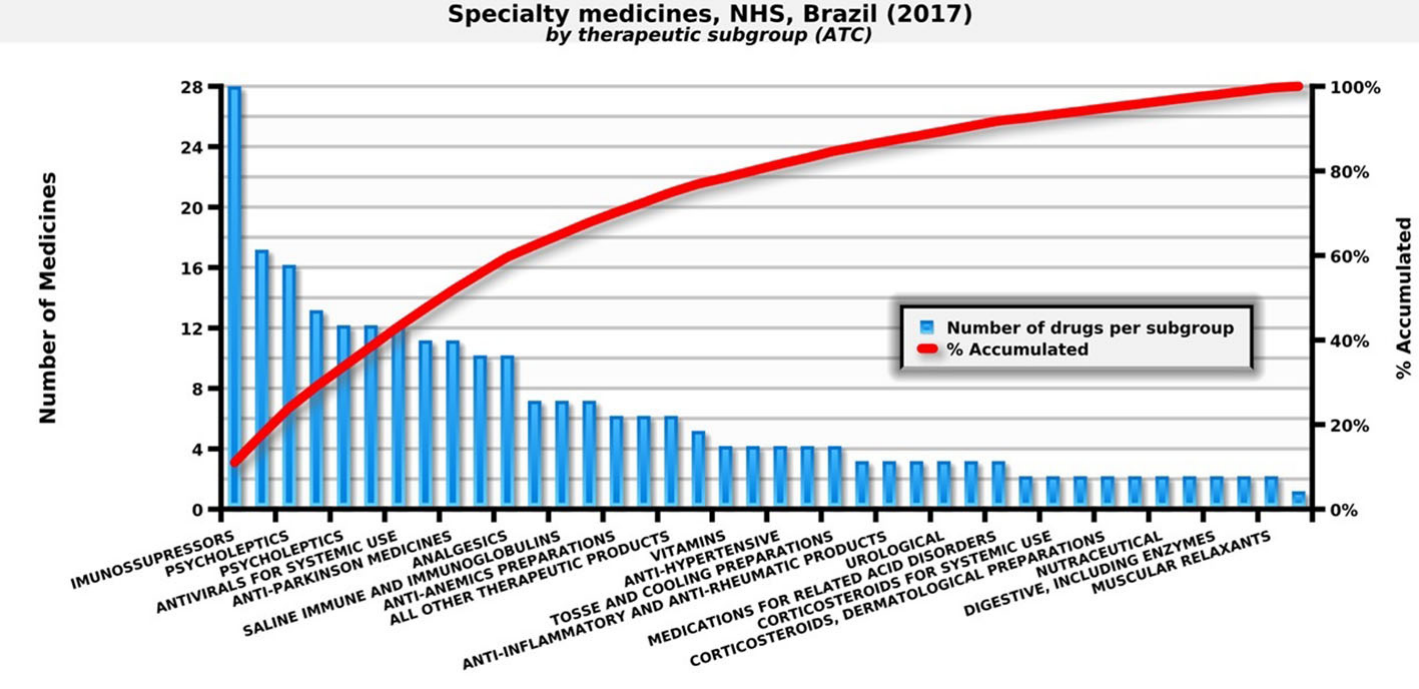
Number of specialty medicines by therapeutic subgroup paid by the NHS. Source: self-elaboration, data available from the NHS (Brasil, 2018b).

In 2017, the NHS in Brazil provided 247 medicines for outpatient medical specialties. These medicines are important due to their epidemiological impact and/or the cost that they represent for families (**Figure 1**).

Alternative strategies adopted by low and middle-income countries (LMICs) for public pharmaceutical supply include central medical stores, autonomous supply agencies, direct delivery systems to patients, and dispensing sites in medical centers/units. Consequently, there will be economic implications of the alternative scenario of the private sector–public sector collaboration model with private community pharmacies accredited by the NHS. Similarly to many developing countries, Brazil adopted a model for the organization, payment, and logistic provision of medicines that was almost completely state-owned. The public sector, in the three levels of management (municipal, state, and federal), adopted a model of payment for inputs/economic resources consumed.

The efforts of Brazil to achieve UHC for medicines resulted in increased public financing of medicines and their availability, ranging from <50 in 1999 to 62% in 2014 (**Table 1**), reducing avoidable hospitalization and mortality. However, the lack of access still remains in Brazil with still lower levels of access compared with other middle-income countries (Holst et al., 2016). Health benefits that have accrued include an increase of 13.8 years in life expectancy since the inception of the NHS in Brazil (IBGE, 2019). However, factors including inequalities in family income, insufficient financing, and cooperation between private and public sectors are still issues that need improvement as these can impact on the quality of life of the Brazilian population (Castro et al., 2019).

**Table 1:** Country income level and access to essential medicines (WHO, 2004).

Country income group	Median reported access level (%)	Minimum reported (%)*	Maximum reported (%)*
Low-income	60	10	93
Middle-income	85	30	100
Brazil 1999	<50	–	–
Brazil 2001 (Castro et al., 2019)	47	41	53
Brazil 2014 (Nascimento et al., 2017)	62	61	64
High-income	100	98	100

*The data from Brazil refer to the mean, and the values for minimum and maximum refer to the confidence interval (95%).

Consequently in this study, we will undertake an economic evaluation to understand the funding needs of the Brazilian NHS in a scenario of adopting a pharmaceutical service organization similar to that seen in many high-income countries, that is, hiring/accrediting private pharmacies to provide medicines access with public funds. As a result, we will seek to appreciably improve access to medicines in Brazil compared with the current situation. At the same time, keep to the concept of no limit on medicine expenditure among families as this is an important concept in Brazil.

## Methods

### Overview

We performed an economic evaluation regarding the provision of medicines with public funds in two alternative scenarios: public pharmacies (NHS state-owned facilities) *versus* private pharmacies (NHS agreements). The analysis assumed the perspective of the NHS. We identified the types of resources consumed and the amounts and value of each item from Brazilian government data sources (Brasil, 2018b). All the monetary values were adjusted according to the purchasing power parity index of the World Bank (The World Bank, 2019).

### Model of Pharmaceutical Services

**Figure 2:**
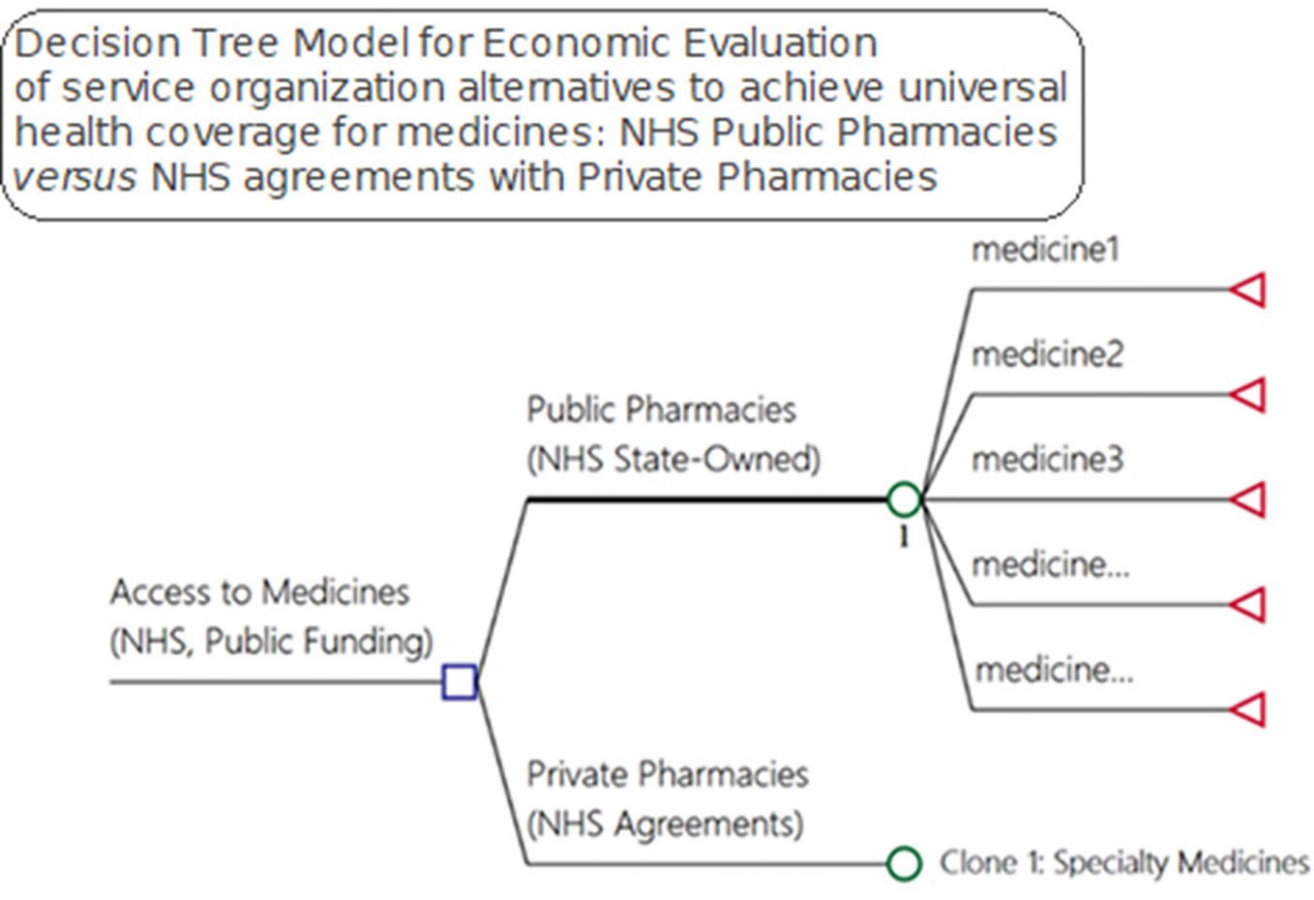
Model for economic evaluation between pharmaceutical service organization alternatives for medicine access, NHS public pharmacies *versus* NHS agreements with private pharmacies—OECD medicines access model).

We developed a model to evaluate alternative pharmaceutical service organization to provide access to medicines within public healthcare systems (**Figure 2**). The decision-tree model used inputs from a pharmaceutical service organization in Brazil for the medicines and supplies for outpatient medical specialties. We considered effectiveness in terms of medicine access as it is one of the goals of NHS funding, with citizens in Brazil having a right to health.

Litigation, direct expenses, hospitalizations, and death can be the consequences of a lack of access to pertinent medicines that can be used to cure, prevent and/or reduce the progression of both infectious and noninfectious diseases. Due to the difficulty in measuring the probabilities for each drug—247 in total—in relation to a given consequence, in our study we investigated and calculated the total cost of each of these outcomes (**Figure 2**) to provide insight into current expenditures for these items.

### Availability of Data About Outcomes: Litigation, Out-Of-Pocket Expenses, Hospitalizations, and Death

In Brazil, NHS Federal expenditures on health litigation are mostly a result of citizens demanding medicines (Conselho Nacional de Justiça, 2019; Machado et al., 2011). We performed a search of the Ministry of Health (MoH) website, available literature, and we contacted the officers at the Department Juridical Affairs of the MoH responsible for providing NHS technical–juridical information in cases of health litigation to gain robust data on the current status of medicine litigation in Brazil.

We obtained data on out-of-pocket expenses (household income and expenditures) on health and medicines from the National Survey by Household Sample (PNAD) for 2017 (IBGE, 2018) and from the last available dataset from Household Budgets Survey (POF) for the years 2008/2009 (IBGE, 2010) for urban and rural areas.

We obtained data related to specialty care with outpatient and hospital admissions due to conditions potentially treatable or avoidable with the use of medicines from Brazilian NHS databases from the frequencies of outpatient and hospital admission services and its costs (payments) for the period from January to December of 2017. Furthermore, we extracted from the National Mortality database (2017) the deaths from potentially preventable causes (Brasil, 2018b).

### Resource Use

**Figure 3:**
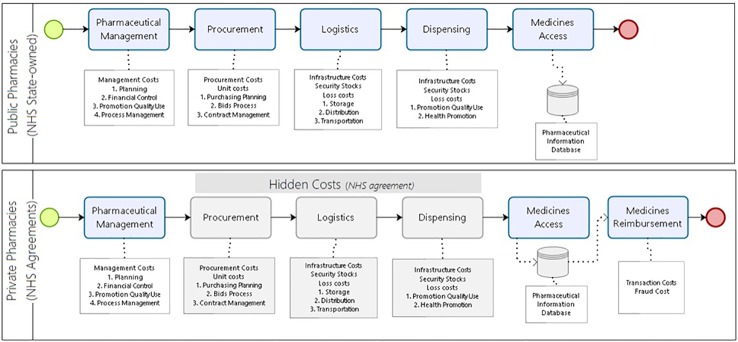
Costs incurred to provide medicine access for the developing country model of pharmaceutical services.

We identified the relevant costs incurred in providing medicine access for the current model of pharmaceutical services in Brazil. In the alternative scenario, all steps for procurement, logistics, and dispensing would be under a hiring agreement between the community private pharmacies and the NHS. The reimbursement happens for each medicine dispensed. The costs related to the management of the program that remain in the control of the NHS (public sector) are equivalent in both models (**Figure 3**).

### Model Inputs

We performed a search of the available data in the MoH administrative databases to obtain details of the medicines dispensed and the amount paid in 2017 (monthly data) for the specialized component of pharmaceutical services. These medicines are of considerable importance because of either their epidemiological impact or their costs. Examples of such medicines include anti-TNF alphas for rheumatoid arthritis, insulins for diabetes, statins for cardiovascular diseases, antivirals for hepatitis, and medicines for schizophrenia (Andrieux-Meyer et al., 2015; Dos Santos et al., 2016; Marra et al., 2017; Barbosa et al., 2018; do Nascimento et al., 2018).

Due to lead times for procurement and logistics, we set the safety stocks in the public sector at four months, which we present as costs with immobilized assets accepting that this four months of safety stocks produces an opportunity cost. In LMICs where inflation and interest rates are usually higher than high-income countries, as seen in Brazil, there are rules recommending NHS managers to invest in low-risk funds when expenditures are not happening. *Logistics operation costs* are functions which include warehousing, inventory management, and transportation in bulk. In LMICs, this part of the logistic process may be executed using public facilities and public servants. To simplify the model, we considered that private companies hired by public administration would undertake bulk pharmaceutical logistics operations as is currently the case among the Ministry of Health and other large Departments of Health in the Brazilian States, which is also the situation in the State of Minas Gerais and Rio de Janeiro. In the logistic agreements made by these States and the Ministry of Health, the costs are usually established as a percentage of the volume of loads and their respective prices. We set the *costs with losses* due to obsolete medicines, those expiring, spoilage, wastage, and theft at five percent of the annual cost of the medicines purchased. This is because the information for pharmaceutical losses is currently uncertain both in the public or private sectors. Consequently, we adopted a wide interval in the sensitivity analysis to assess these influences in the different scenarios.

The scenario of private pharmacy hiring agreements for dispensing transfers the steps related to the procurement, logistics operations, and dispensing to the private sector. The NHS pays private pharmacies for access to each drug dispensed and must cover the cost of outsourced steps. The economic risks of these operations are implicit and under the responsibility of the private sector. All the parameters as a result of the literature searches, NHS databases, surveys of government websites and information provided by public authorities adopted as model inputs, are summarized in **Table 2**.

**Table 2:** Parameters, base value, and intervals adopted as model inputs for pharmaceutical dispensing services NHS.

	Parameters	n	Cost US$PPP	Interval	Total	Source
	**Medicines purchased by NHS**	244	NHS Data	*		Brasil, 2018b
	**Medicines costs private pharmacies**	244	PMVG 0%	**		Brasil, 2018a
**Dispensing and maintenance costs of public pharmacies by NHS**	**Salaries and wages expenses **					
	Pharmacists, monthly salary	9	5,000	3,192–8,865	45,008	EPSM, 2017
	Attendants, monthly salary	92	2,268	1,570–4,015	208,647	EPSM, 2017
	**Facilities structure expenses**					
	Operation services hours	12 h/day; 5 days/week; 22 days/month				Survey
	Rent/property (real state value, m2)	1	60	54–65	60	FIPE, 2019
	Depreciation rate (equipment’s, 5%)	1	172,467	155,220–189,713	172,467	Minas Gerais, 2017
	Utilities (telecom, energy, water)	1	25,000	22,500–27,500	25,000	Minas Gerais, 2017
	Security, cleaning services and supplies	1	15,000.00	13,500–16,500	15,000	Minas Gerais, 2017
	Office supplies	1	10,000	9,000–11,000	10,000	Minas Gerais, 2017
	**Number of dispensing per month**	44.000		11.000–88.000		Minas Gerais, 2017
	**Number of medicines per patient**	2.4		1.3–3.8		Cunha et al., 2002; Lima et al., 2017; Santos and Nitrini, 2004
	**Costs immobilized assets**	0.0971		0.0620–0.1373		Survey
	**Logistics operation costs**	4%		2–6%		
	**Costs with losses**	5% annual volume handled		2–40%		Minas Gerais, 2017
**Benefits**	**Costs with potential frauds in reimbursement**	2%		0.5–5%		Brasil, 2011
	**Medicines availability NHS**	65%		55–75%		Guerra, 2004; Lima et al., 2017
	**Medicines availability Private Pharmacies**	90%		85-95%		Guerra, 2004; Nascimento et al., 2017

*Consulted data from the acquisitions made in NHS during the year 2017.

**The reference value: defined as the value of the brand at the 1st decile price, for government purchases without taxes (PMVG 0%, CMED).

### Dispensing and Maintenance Costs of Public Pharmacies by NHS

To develop the model, we considered public pharmacy operation hours as twelve hours of service per day, five days a week and twenty-two days a month. To establish these values, we surveyed the webpages of the twenty-seven Brazilian States of the NHS pharmaceutical dispensing services. For public employees, we considered 8 h per day and 40 h per week. This is because the NHS State pharmacies, which have the responsibility for dispensing specialty medicines, do not open on Saturdays and Sundays. We obtained the number of medicines dispensed per month and the other values of production of a typical public pharmacy for specialty medicines, in the NHS databases in the State of Minas Gerais. The costs related to the real estate were valued based on the cost per m^2^ for renting from the national index of economic research (FIPE, 2019). The values of the public employees’ salaries for Brazil were obtained from the market signals research station—EPSM (EPSM, 2017).

The average monthly costs of dispensing and maintaining an NHS public pharmacy are the result of the sum of all the resources spent on producing the service divided by the number of dispensing per month:

$$\mathrm{Average Cost per Dispensing}=\frac{\mathrm{Resources Consumed}}{\mathrm{number of dispensation realized in}\;30\;\mathrm{days}}$$

### Unit Cost of Medicines Purchased by NHS

We retrieved the unit cost of the specialty medicines provided by NHS from the official data bank of medicine procurement for the year of 2017 (Brasil, 2018c). This is the real-world price for the NHS as Brazilian public health authorities cannot currently make confidential price agreements under the law.

### Unit Cost of Medicines in the Private Pharmacies

In Brazil, the National Pharmaceutical Market Authority (CMED) regulates the maximum prices for government and consumer purchases. This data is updated monthly by CMED and made available for public consultation on the government website (Brasil, 2018a). Using this information, we created a database with all prices for all the brands available for the specialty medicines. We used the price list released by CMED for September 2018.

We extracted the unit cost of each specialty medicine from the database created for all brands available in the Brazilian market for the selected medicines. The unit cost for private community pharmacies has been defined as the maximum medicine price for the government purchases (PMVG) situated in the first decile of all brands available. We inputted in the model parameters for potential market prices for NHS payment in the private pharmacies using the following rational that was previously adopted by the Ministry of Health initiative called “Aqui Tem Farmácia Popular do Brasil”:

- *Minimum value*—cheapest brand between all medicines in the market for each drug;
- *Base value*—brand price in the first decile of all medicines in the market for each drug;
- *Maximum value*—brand price in the median of all medicines for each drug.

### Monthly Treatment Cost

We estimated the monthly costs for each medicine by multiplying the unit cost by the number of pharmaceutical units required for a monthly treatment (30 days). For these estimates, we considered the number of pharmaceutical units needed for each treatment based on Defined Daily Doses (DDD) from the WHO Collaborating Centre for Drug Statistics Methodology (WHO, 2018). The DDD is the assumed average maintenance dose per day for a drug used for its main indication in adults. In case information was not available in the ATC/DDD system, the maximum amounts permitted and authorized by the Ministry of Health for dispensing, according to the main indication recommended in the Clinical Protocols and Therapeutic Guidelines adopted in the NHS, were used to calculate the DDDs (Brasil, 2019).

We added to the monthly treatment costs the sum of the dispensing and maintenance costs in the public pharmacies, the logistic operation costs, and any losses per month. In the private model, the monthly treatment cost additionally included the percentage costs with losses from potential frauds in reimbursement as stated in audit reports (**Figure 3**).

### Potential Benefits

The increase in the availability of medicines incorporated routinely into the Brazilian NHS should help reduce litigation and out-of-pocket expenses as well as subsequent hospitalizations and deaths (outcomes). The medicine access research reports (2004 and 2017) showed a 62% availability of medicines, while in the private pharmacies this was approximately 90%. We assumed an access rate in public pharmacies of 65%, ranging from 55 to 75%. For the private model, we assume 90% access, ranging from 85 to 95%.

### Sensitivity Analysis

We performed a sensitivity analysis to test uncertainties arising from the model inputs. As the first step, we tested univariate variables to assess how the results might change. After testing each parameter individually, we evaluated the impact on results through probabilistic sensitivity analysis using Monte Carlo simulation.

## Results

**Figure 4:**
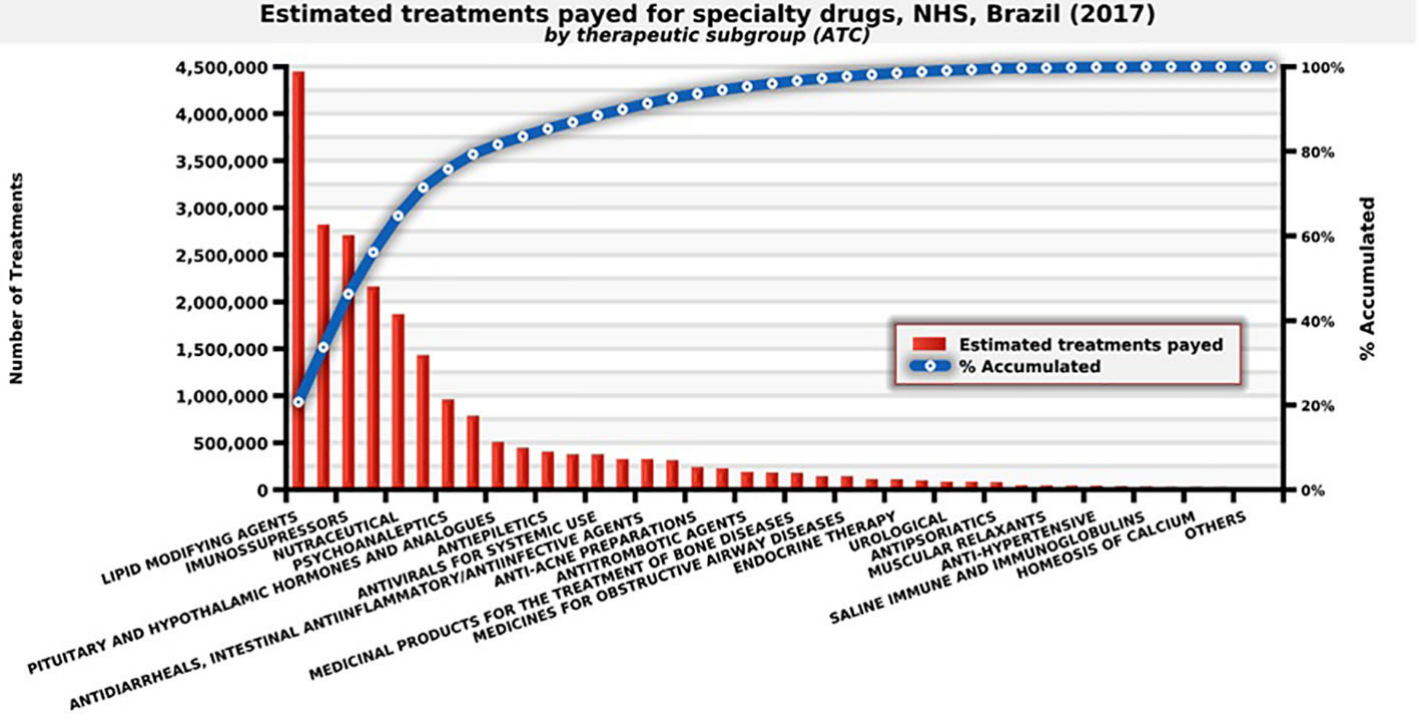
Number of estimated treatments for each medicine provided by the therapeutic subgroup, NHS, Brazil (2017). Source: self-elaboration, data available from NHS (Brasil, 2018b).

NHS distributed approximately 21.4 million treatments in Brazil in 2017, spending eight billion dollars. Examples include immunosuppressant for rheumatoid arthritis, statins for cardiovascular diseases, antivirals for hepatitis, and mental health medicines used for schizophrenia (**Figure 4**).

**Figure 5:**
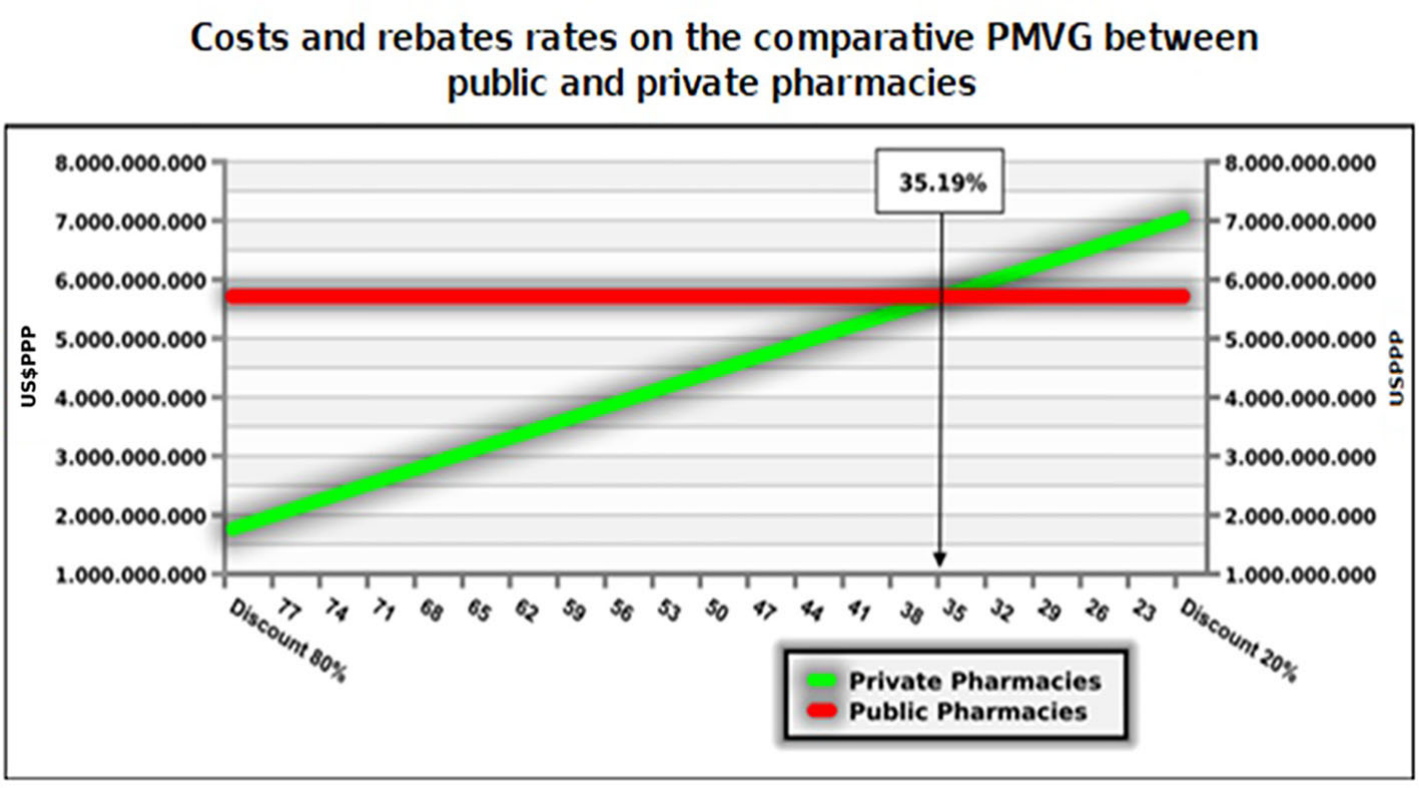
Costs and rebates rates on the comparative PMVG between public and private pharmacies.

The model estimated an incremental cost of US$3.1 billion (DPPP) in a scenario of no rebates over medicine reference prices for government purchases (PMVG) but with an increase in the average availability of medicines from 65% to 90% for citizens across the country and for all classes of family incomes (**Table 2** and **Figure 5**). The scenarios above 35% rebates put the alternative of hiring private pharmacies as dominant. Higher rebates are feasible and may lead to savings of US$1.3 billion (PPP) per year (**Figure 5** and **Table 3**).

**Table 3:** Forecast incremental cost in different rebate scenarios for distribution of medicines in private pharmacies.

Strategy	Cost (Billions US$PPP)	Incremental Cost (Billions US$PPP)
**Forecast scenario: no rebate**		
Public Pharmacy (State-owned facilities)	5,711,682,916.31	
Private pharmacy (NHS agreements)	8,812,681,695.98	3,100,998,779.67
**Forecast scenario: rebate 30%**		
Public Pharmacy (State-owned facilities)	5,711,682,916.31	
Private pharmacy (NHS agreements)	6,168,877,187.19	457,194,270.88
**Forecast scenario: rebate 50%**		
Public Pharmacy (State-owned facilities)	5,711,682,916.31	
Private pharmacy (NHS agreements)	4,406,340,847.99	(- 1,305,342,068.32)

### Funding Demanded to Achieve Universal Health Coverage

To understand the funding demanded to achieve UHC for medicines, we analyzed the budget impact forecast scenario with 35.19% rebates over the first decile brand prices. In this scenario, we may see the estimated cost of hiring private community pharmacies. We estimate the incremental budget, considering that between the first and second years there would be a further 25% increase in access in the scenario of the NHS choosing to hire private pharmacies. The budget impact for the NHS would increase at a rate of 6% after that, which is the average growth of the last decade of the dispensing of these medicines in the NHS (**Table 4**). The impact of incremental funding is related with the access gain of 25% (second year) to move from payment for inputs of public dispensing to the private pharmacies contracting, and the incremental budget in five years would be US$4.8 billion PPP. We have yet to explore what would be the reduction in hospital/outpatient costs and lawsuits, with the yearly expenses for the NHS at US$9 billion and US$1.4 billion PPP, respectively in 2017.

**Table 4:** Funding demanded to achieve universal health coverage for medicines presented as budget impact analysis, estimate for five years forecast scenario with 35.19% rebates.

Year	Treatments		Access Growth	Budget Estimate Impact	Budget Estimate Impact
	Public	Private	(%)	Public Pharmacies	Private Pharmacies^b^
1	21,881,719.30	21,881,719.30	base year	5,711,682,916.31	5,711,683,192.33
2	23,174,852.21	27,352,149.13	25^a^ + 6	6,049,223,355.86	7,139,603,991.57
3	24,544,404.75	28,968,565.26	6	6,406,711,254.25	7,561,529,559.43
4	25,994,893.03	30,680,505.93	6	6,785,325,430.57	8,008,389,452.70
5	27,531,100.10	32,493,616.29	6	7,186,314,382.00	8,481,657,198.57
***Incremental Access***		***18,249,586.52***	***Incremental Budget***		***4,763,606,055.61***

^a^25% is the incremental access between expected availability scenario of hiring private pharmacies. ^b^The reference value: defined as the value of the brand at the 1st decile price, for government purchases without taxes (PMVG 0%, CMED), with a rebate rate of 35.19%.

### Sensitivity Analysis

We perform the univariate sensitivity analysis on the variables with uncertainty and potential impact on the results. These included the monthly salary of pharmacists, the number of pharmacists per pharmacy, the number of medicines dispensed per month, the average number of medicines prescribed per patient, the unit cost of medicines, as well as the cost associated with logistic losses and losses due to reimbursement (**Table 5**).

**Table 5:** Univariate sensitivity analysis of the parameters.

	Parameter	Base value	Interval	Variation of incremental cost (PPP$)
**Public Pharmacies**	**Monthly cost of pharmacist (salary, US$PPP)**	5,000.83	3,191.48 8,865.24	3,104,313,696.20 3,093,918,801.60
	**Number of pharmacists per pharmacy**	9	1 16	3,109,142,800.33 3,093,872,761.60
	**Number of dispensing per month**	44,000	11,000 88,000	2,902,559,701.36 3,134,071,959.39
	**Number of medicines per patient**	2.4	1.3 3.8	3,045,028,783.23 3,125,368,491.05
	**Costs with losses**	1.05	1.02 1.4	3,257,656,501.96 1,273,325,352.95
**Both**	**Coefficient of variation of the Unit Cost**	1	0.35 1.65	1,042,354,439.25 5,159,643,120.10
**Private Pharmacies**	**Costs with potential frauds in reimbursement**	1.02	1.005 1.05	2,984,393,133.56 3,334,210,071.90

The sensitive analysis scenarios demonstrated that the two most important parameters that might affect the incremental costs are the coefficient of variation of the unit costs and the cost associated with losses. Increased logistic efficiency of the NHS, with reduced losses, raises the incremental cost to hire private pharmacies by 5.1%.

Nevertheless, in a scenario of lack of control and increased losses, the incremental cost to hire private pharmacies would reduce by 58.9%. In a scenario of reduced unit costs by 65%, with a larger share of generic/similar medicines, for example, there would be a reduction of the incremental cost to hire private pharmacies by 66.4%. Whereas, if there is an increase in the average unit cost due to lower supply/share of generic/similar medicines, this may produce an increase in the incremental costs to hire private pharmacies by 66.4%.

### Model Limitations

**Figure 6:**
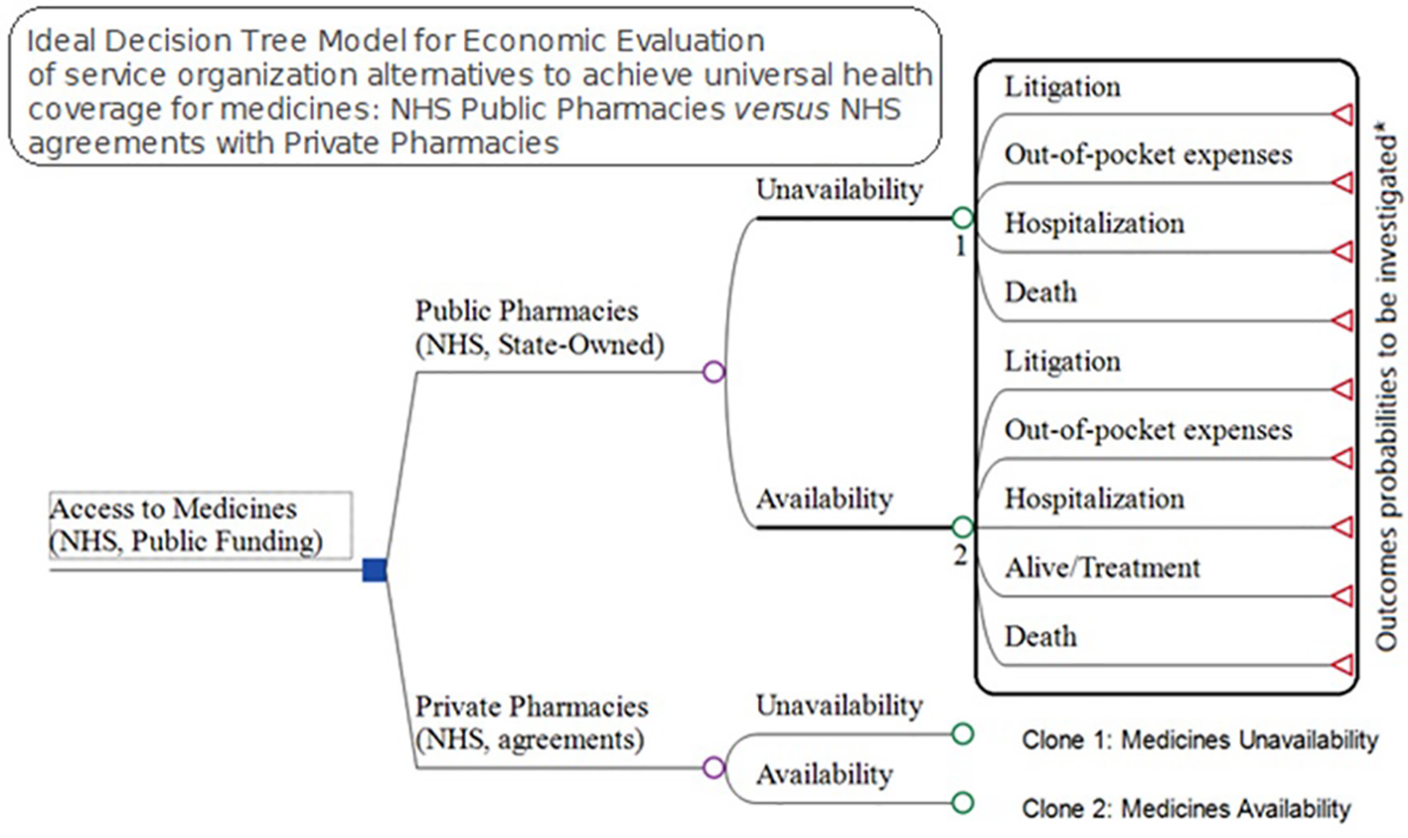
Ideal Model for economic evaluation between pharmaceutical service organization alternatives for medicines access, NHS public pharmacies *versus* NHS agreements with private pharmacies.

Understanding that the lack of medicine access may have consequences including litigation, out-of-pocket expenses, hospitalizations, and death, we designed an ideal model to try and incorporate these parameters. However, the probabilities for each of 247 specialty drugs provided by the NHS in 2017 for these four consequences are difficult to measure. Consequently, in our study, we just investigated the total cost for each of these outcomes once the expenses for the NHS and families are linked to the lack of access, excluding premature deaths (**Figure 6**).

### Potential Benefits

**Figure 7:**
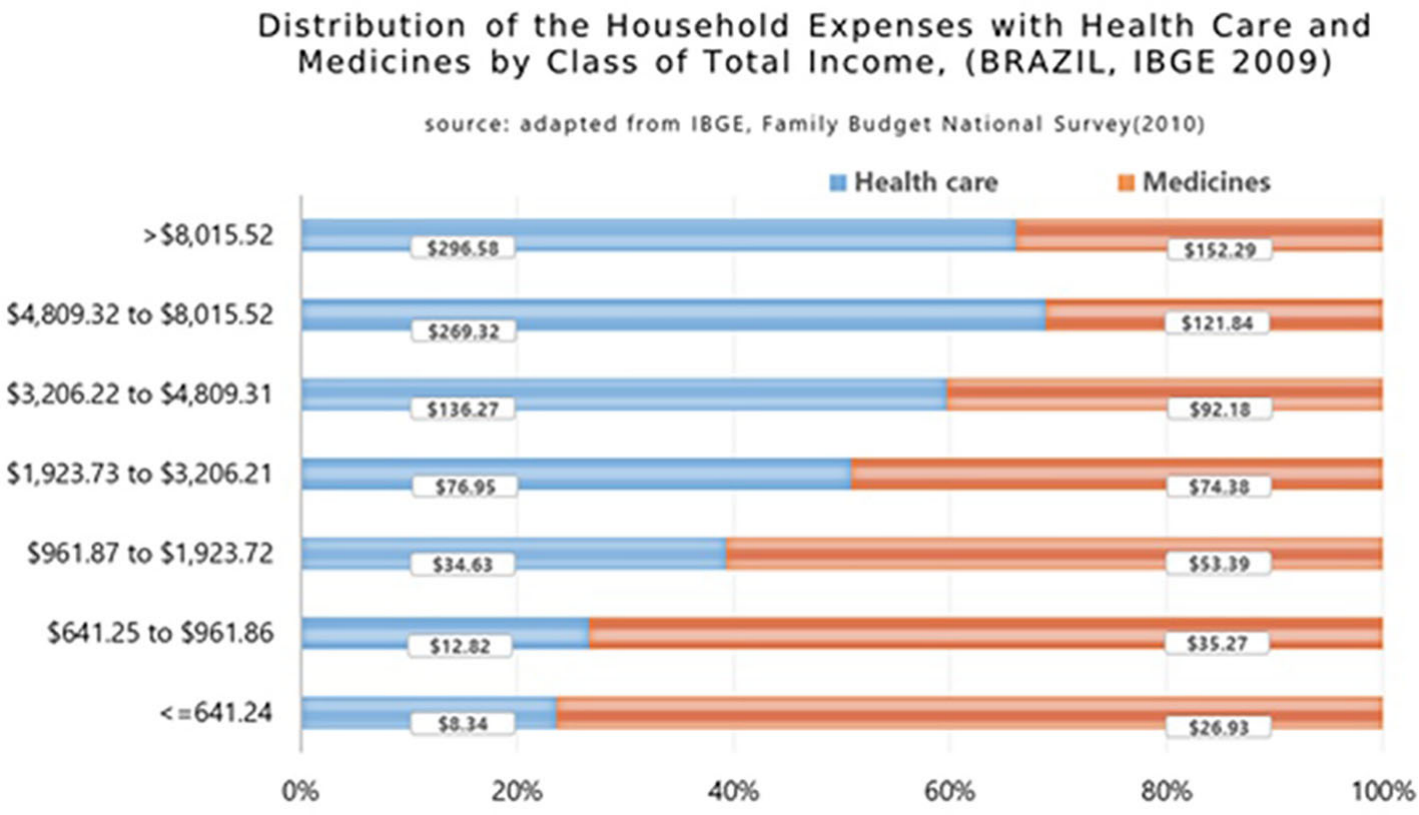
Distribution of the household expenses with health care and medicines by class of total income in Brazil (2009) (Garcia et al., 2013).

Data from the Family Budget Survey (POF) showed that the poorest Brazilian families medicines’ expenditure was 66.5% of their total health spending (IBGE, 2010; Marra et al., 2017). The POF registered important differences between the distributions of household expenditures with healthcare and medicines concerning the family income distribution of the Brazilian population by total income class (**Figure 7**) (Sindusfarma, 2018). The data shows that the lower the income, the higher the impact of spending on medicines as a percentage of total family expenses on health. The poorest families, which represent 22.5% of the country, have a burden of more than 76% of their expenses on medicines in relation to their total health care costs, while in the upper levels of the Brazilian society this reduces to 34%.

The combined scenario of low availability of medicines and economic distress related to health expenses can lead to litigation against the NHS. As previously mentioned, in Brazil, NHS Federal expenditures on litigation are mostly due to citizens demanding medicines, corresponding to approximately 80% of the total lawsuits. In dollar amounts, as mentioned, the NHS currently spends approximately US$1.4 billion (PPP) per year on litigation. This is in addition to the costs associated with regular administrative claims for medicines received by the NHS (Conselho Nacional de Justiça, 2019).

**Figure 8:**
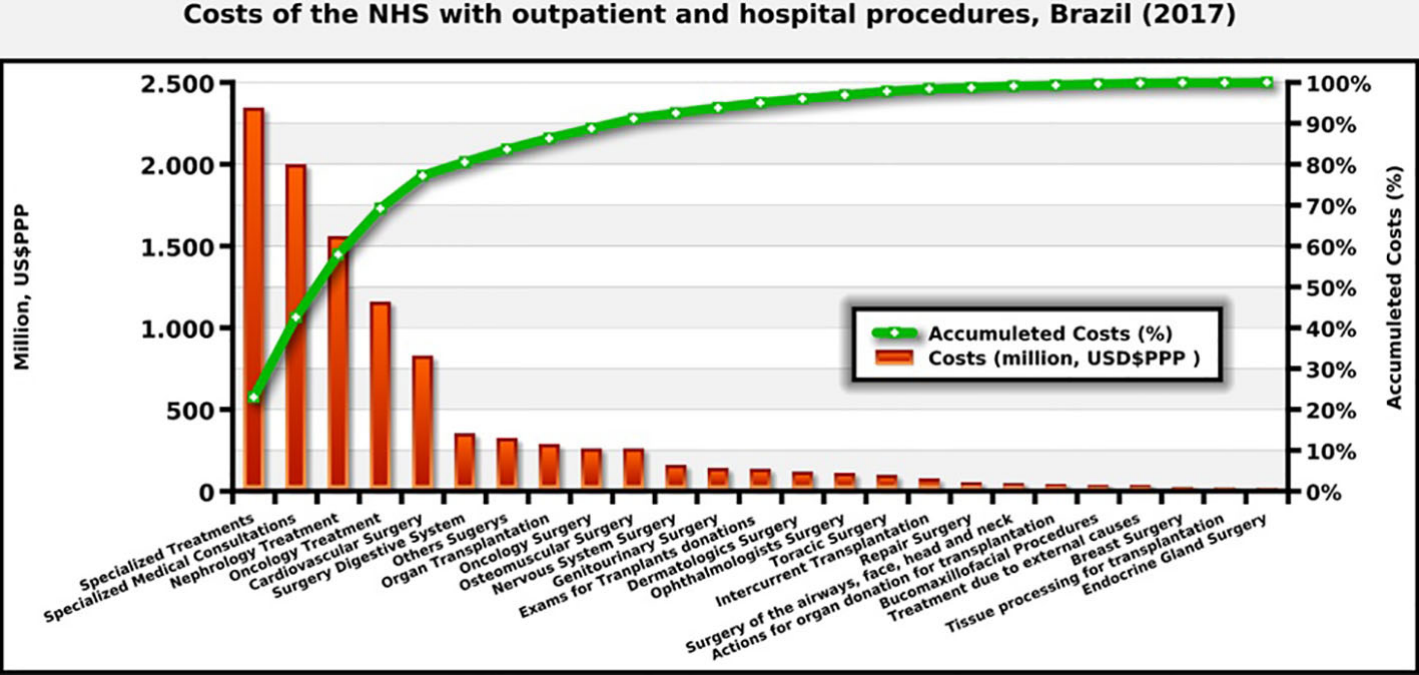
Costs of the NHS with outpatient and hospital procedures in Brazil (2017).

In 2017, data from DATASUS indicate that the NHS in Brazil paid for more than 2.3 million hospital admissions and 5.6 million outpatient care procedures associated with procedures and complications potentially related to the treatments for which clinical protocols in the NHS exist. The care provided by the NHS\ for these procedures, as mentioned, costs approximately US $9 billion PPP in 2017 (**Figure 8**). The most demanded treatments were related to kidney problems, cardiovascular disease, infections and psychiatric treatment, for which there are medicines listed by the NHS for the control and a reduction in the worsening of the clinical conditions.

**Figure 9:**
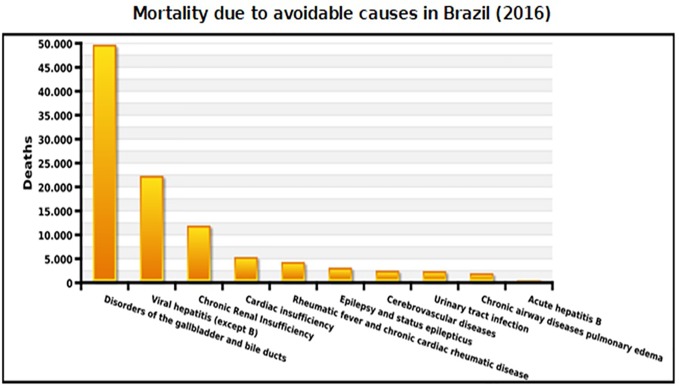
Mortality due to avoidable causes in Brazil (2016).

The last year with available data for mortality is 2016. The number of deaths from potentially preventable causes, including adequate clinical management and medicine usage, was more than 100,000 cases (**Figure 9**). Deaths related to hepatic, renal, cardiovascular and infection problems were highlighted, and most of these clinical conditions are treatable and/or preventable with medicines incorporated in the Brazilian NHS, which should be readily available.

### Proposed Private Sector–Public Sector Collaboration for Pharmaceutical Service Organization

**Figure 10:**
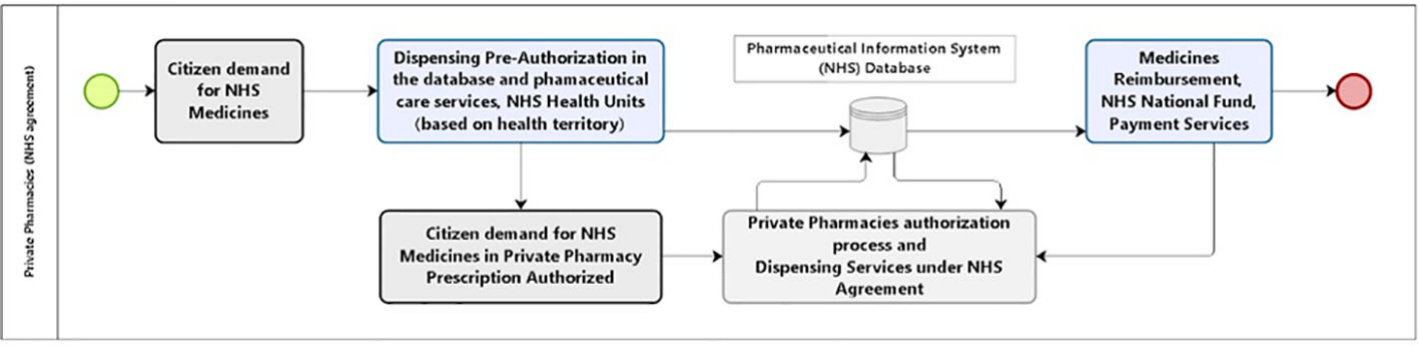
NHS service model to provide access to medicines, private pharmacies agreements.

The optimal private sector–public sector collaboration looks to be the key to achieving UHC for medicines reducing subsequent avoidable hospitalization and mortality as well inequalities among families concerning household expenses. The resultant service organization (**Figure 10**) might consider the use of an information system and a simple workflow. It is important to preserve some of the NHS advantages of having the citizens geographically referred to the public health service centers in their neighborhood. The available health centers provide medical care, prescriptions, and authorization to obtain medicines in a private pharmacy of the citizen’s choice. Pharmacists and pharmacies should work under the same information system and send invoices for payment to the appropriate government agency as typically happens in high income countries. The authorization system of the NHS would validate and reimburse the medicines (**Figure 10**).

## Discussion and Conclusions

Despite important advances made in the implementation and improvement of NHS in Brazil, access to medicines remains a focus of inequality and health litigation. Insufficient progress though has been made about the direct disbursement of the poorest families with pharmaceutical products over the years.

The total value of medicines purchased annually by the NHS should place it in a position of obtaining appreciable rebates. The scenarios above 35% rebates put the alternative of hiring private pharmacies as dominant, but higher rebates are feasible and may lead to savings of US$1.3 billion per year (DPPP) (**Figure 5** and **Table 3**). This study does not recommend the adoption of reference pricing for government purchases (PMVG with 0% tax) as a basis for NHS payment for dispensing. This is according to data from the Union of the Pharmaceutical Manufacturer Companies (Young et al., 2017) which state that in the Brazilian pharmaceutical market there is an average rebate of 41% over the unit cost of medicines. This can potentially be higher with more aggressive purchasing approaches. Reference pricing may reduce the size of the rebate alongside other concerns with reference pricing including appropriate comparator countries and their economic considerations including GDP (Young et al., 2017; Mahlich et al., 2019). There are also issues of transparency with reference pricing since list prices within countries typically do not include confidential discounts, which are now a key element of pricing negotiations for medicines across countries (Ferrario et al., 2017; Vogler et al., 2017; Maskineh and Nasser, 2018; Robinson et al., 2018).

Preventable morbidity and mortality due to a lack of access to medicines affects developing countries' economies such as Brazil. In addition to the social and economic consequences, the inefficiency of the model adopted leads to productivity loss for families, especially low-income families who are affected by economic crises and usually have to accept working conditions not protected by social security or labor legislation. It is possible that the consequences of this potentially preventable morbidity and mortality affect the economy as a whole. The seriousness of this framework implies an increase in costs for the NHS itself due to clinical complications and, finally, loss of quality of life for the citizens. It would be worth pointing out that the loss of citizens' quality of life alone would be reason enough to justify the improvement of public health policies and the reduction of inequality in access to medicines in the country.

As mentioned, the economic advantage in the private model occurs with a rebate rate of 35%. The new model proposal (**Figure 10**) considers the use of an information system and a simple workflow. Citizens would still be referred to their health territory where they may receive medical care, prescriptions, and authorization to obtain prescribed medicines in the private pharmacy of their choice. Pharmacies would send the invoice for payment to the appropriate government agency. The authorization system of the NHS would subsequently validate and reimburse the medicines.

The implementation studies necessary to change the actual service models to provide access to medicines are complex, and the success or failure will rely on the strategies adopted. This implementation phase will demand specific studies which were not the focus of this paper. Overall, we believe the results of this economic evaluation robustly demonstrate the benefits of the introduction of an optimal model of collaboration between public and private sectors. The current model of pharmaceutical services provided by NHS Brazil produces inequality, shortages of medicines especially for chronic diseases, and jeopardizes other health policies and household income. The model used in many high-income countries with accredited private community pharmacies appears more economical alongside competent price negotiations for medicines which we believe is feasible. The results also suggest that the inclusion of private community pharmacies in NHS has the potential for improving access to medicines by 25% across all income classes with the greatest benefit to the poorest families.

Overall, we believe that the results presented in this article can result in new health policies and help to reduce inequalities in access to medicines in Brazil, with potential savings in lives and public resources through the negotiation of prices practiced in the Brazilian market. In addition, the study helps support decision making by managers by allowing them to evaluate different scenarios within the Brazilian context. We will be monitoring these developments in the future.

## Data Availability Statement

All datasets generated for this study are included in the article/**Supplementary Material**.

## Author Contributions

All authors contributed to the planning, analysis, and interpretation of data, and preparation and approval of the final version of the manuscript.

## Conflict of Interest

The authors declare that the research was conducted in the absence of any commercial or financial relationships that could be construed as a potential conflict of interest.
