# Supplementary material for: Funding and Service Organization to Achieve Universal Health Coverage for Medicines: An Economic Evaluation of the Best Investment and Service Organization for the Brazilian Scenario
Source: Front Pharmacol. 2020 Apr 14;11:370. doi: 10.3389/fphar.2020.00370 (PMC7175689; doi:10.3389/fphar.2020.00370)
Supplement: Supplementary file 1 [file DataSheet_1.docx]

**Appendix I**

Conditions potentially treatable or avoidable

- Collection and exams for donation of organs, tissues and cells and transplantation
- Tissue processing for transplantation
- Endocrine gland surgery
- Transplantation of organs, tissues and cells
- Pre and post-transplant follow-up and intercurrences
- Thoracic surgery
- Surgery of the central and peripheral nervous system
- Surgery of the upper airways, face, head and neck
- Treatment in nephrology
- Surgery of the circulatory system
- Surgery of the digestive tract, attached organs and abdominal wall
- Osteomuscular system surgery

**Appendix II** – Medicines Unit Prices in Brazilian Currency (BRL, Reais)

1. NHS unit prices, public purchases (NHS Databases).

2. The reference value: defined as the value of the brand at the 1st decile price, for government purchases without taxes (PMVG 0%, CMED)

| **Medicines** | **NHS Unit Prices (BRL)** | **Market Unit Prices (BRL)** |
| --- | --- | --- |
| Abatacepte 250mg Frasco Ampola | 902,2377 | 1131,85 |
| Acetazolamida 250mg Cpr | 0,358 | 0,3028 |
| Acido Nicotinico 500mg Cpr | 1,0168 | 0,8977 |
| Acido Nicotinico 750mg Cpr | 1,0435 | 1,0723 |
| Acitretina 10mg Cpr | 2,17 | 2,9143 |
| Acitretina 25mg Cpr | 5,31 | 7,0687 |
| Adalimumabe 40mg Seringa Preenchida | 2012,4 | 2490,94 |
| Adefovir 10mg Cpr | 6,28 | 20,5993 |
| Alfacalcidol 0,25 mcg Cpr | 1,21 | 1,5007 |
| Alfacalcidol 1mcg Cpr | 2,77 | 3,793 |
| Alfadornase 2,5mg Ampola | 71,59 | 47,6633 |
| Alfaepoetina 10000UI Frasco Ampola | 23,07 | 54,031 |
| Alfaepoetina 1000UI Frasco Ampola | 7,13 | 6,0625 |
| Alfaepoetina 2000UI Frasco Ampola | 12,4692 | 15,4158 |
| Alfaepoetina 3000UI Frasco Ampola | 5,62 | 57,5665 |
| Alfaepoetina 4000UI Frasco Ampola | 18,3675 | 82,36 |
| Alfainterferona 2b 10000000UI Frasco Ampola | 158,3593 | 108,84 |
| Alfainterferona 2b 3000000UI Frasco Ampola | 31,6651 | 31,77 |
| Alfainterferona 2b 5000000UI Frasco Ampola | 50,9236 | 53,55 |
| Alfapeginterferona 2a 180mcg Seringa Preenchida | 1099,245 | 1300,6 |
| Alfapeginterferona 2b 100mcg Frasco Ampola | 981,86 | 998,52 |
| Alfapeginterferona 2b 120mcg Frasco Ampola | 1177,77 | 1215,34 |
| Alfapeginterferona 2b 80mcg Frasco Ampola | 806,69 | 1457,84 |
| Alfataliglicerase 200U Frasco Ampola | 1258,39 | 1049,31 |
| Amantadina 100mg Cpr | 0,33 | 0,42 |
| Ambrisentana 10mg Cpr | 25,24 | 76,1137 |
| Ambrisentana 5mg Cpr | 25,24 | 38,0567 |
| Atorvastatina 10mg Cpr | 0,2695 | 0,975 |
| Atorvastatina 20mg Cpr | 0,3375 | 0,975 |
| Atorvastatina 80mg Cpr | 0,9764 | 2,073 |
| Azatioprina 50mg Cpr | 1,3731 | 1,0224 |
| Betainterferona 1a 12000000UI Seringa Preenchida | 164,79 | 952,685 |
| Betainterferona 1a 6000000UI (22mcg) Seringa Preenchida | 145,95 | 952,685 |
| Betainterferona 1a 6000000UI 30mcg Seringa FCP | 420,05 | 1736,27 |
| Betainterferona 1b 9600000UI 300mcg Frasco Ampola | 83,2746 | 203,4322 |
| Bezafibrato 200mg Cpr | 0,5701 | 0,434 |
| Bimatoprosta 0,3mg/mL Soluçao oftalmica Frasco | 29,66 | 37,566 |
| Bosentana 125mg Cpr | 9,4344 | 37,0058 |
| Bosentana 62,5mg Cpr | 9,398 | 18,5028 |
| Brimonidina 2,0mg/mL Solucao Oftalmica Frasco de 5mL | 4,7173 | 4,688 |
| Brinzolamida 10mg/ml Suspensao Oftalmica | 36,2952 | 6,94 |
| Bromocriptina 2,5mg Cpr | 1,38 | 1,9354 |
| Budesonida 200mcg Aerossol Bucal | 0,2478 | 0,2947 |
| Budesonida 200mcg Cpr Inalante | 0,2895 | 0,2947 |
| Budesonida 400mcg Cpr Inalante | 0,5653 | 0,5127 |
| Cabergolina 0,5mg Cpr | 16,66 | 13,8612 |
| Calcipotriol 50mcg Bisnaga | 45,4476 | 46,08 |
| Calcitonina 200UI - 100UI Solucao Injetavel | 20,0199 | 27,45 |
| Calcitonina 200 UI - Spray Nasal Frasco | 27,6317 | 56,39 |
| Calcitriol 0,25mcg Cpr | 0,8052 | 1,192 |
| Calcitriol 1,00 mcg Solução Injetável Ampola | 11,9 | 14,73 |
| Certolizumabe Pegol 200mg/mL Seringa Preenchida | 469,8012 | 630,96 |
| Ciclofosfamida 50mg Cpr | 0,5602 | 0,6554 |
| Ciclosporina 100mg Cpr | 3,2894 | 5,0698 |
| Ciclosporina 100 mg/mL - Solução Oral Frasco com 50mL | 3,2318 | 5,1792 |
| Ciclosporina 25mg Cpr | 0,8883 | 1,4918 |
| Ciclosporina 50mg Cpr | 1,9505 | 2,9838 |
| Cinacalcete 30mg Cpr | 13,29 | 441,83 |
| Cinacalcete 60mg Cpr | 26,58 | 864,09 |
| Ciprofibrato 100mg Cpr | 0,3616 | 1,279 |
| Ciproterona 50mg Cpr | 0,51 | 2,5 |
| Custo Unitário SUS- Clobazam 10mg Cpr | 0,2515 | 0,3075 |
| Clobazam 20mg Cpr | 0,4233 | 0,5475 |
| Clobetazol 0,5 mg/g creme | 0,1896 | 0,2727 |
| Clobetazol 0,5mg/g Solucao | 0,06271 | 0,2068 |
| Clopidogrel 75mg Cpr | 0,36 | 1,9954 |
| Cloroquina 150mg Cpr | 0,35 | 0,0676 |
| Clozapina 100mg Cpr | 1,48 | 2,506 |
| Clozapina 25mg Cpr | 0,3559 | 0,6229 |
| Codeina 30mg Cpr | 0,5549 | 0,708 |
| Codeína 3 mg/mL - Solução Oral Frasco 120mL | 0,1937 | 0,2025 |
| Codeina 60mg Cpr | 0,92 | 1,1763 |
| Complemento Alimentar p/ Fenilcetonúricos (pacientes maiores de 01 ano) | 220 | 220 |
| Complemento Alimentar p/ Fenilcetonúricos (pacientes menores de 01 ano) | 175,38 | 175,38 |
| Daclatasvir 30mg Cpr | 39,7591 | 728,3 |
| Daclatasvir 60mg Cpr | 79,23 | 415,8314 |
| Danazol 100mg Cpr | 1,38 | 1,8392 |
| Danazol 200mg Cpr | 2,59 | 3,628 |
| Deferasirox 125mg Cpr | 12,84 | 18,0793 |
| Deferasirox 250mg Cpr | 27,33 | 36,1586 |
| Deferasirox 500mg Cpr | 54,29 | 72,3179 |
| Deferiprona 500mg Cpr | 6,16 | 8,2448 |
| Desferroxamina 500mg Injetavel Frasco Ampola | 14,06 | 19,44 |
| Desmopressina 0,1mg/mL Solucao Nasal | 26,584 | 34,024 |
| Donepezila 10mg Cpr | 0,3565 | 2,2473 |
| Donepezila 5mg Cpr | 0,1635 | 2,0282 |
| Dorzolamida 20mg/mL Solucao Oftalmica | 2,4417 | 5,27 |
| Entacapona 200mg Cpr | 1,71 | 2,1607 |
| Entecavir 0,5mg Cpr | 8,54 | 19,6617 |
| Entecavir 1mg Cpr | 12,879 | 19,6617 |
| Etanercepte 25mg Frasco Ampola | 606,36 | 586,0025 |
| Etanercepte 50mg Frasco Ampola | 1235,3863 | 1132,1325 |
| Etossuximida 50 mg/mL (Frasco de 120 mL) | 0,172 | 0,2128 |
| Everolimo 0,5mg Cpr | 10,9902 | 8,5937 |
| Everolimo 0,75mg Cpr | 15,769 | 12,891 |
| Everolimo 1mg Cpr | 20,9912 | 17,188 |
| Fenofibrato 200mg Cpr | 0,6461 | 1,273 |
| Fenofibrato 250mg Cpr | 1,1134 | 1,487 |
| Fenoterol 100 mcg Aerossol Frasco 200 doses | 0,0455 | 1,141 |
| Filgrastim 300mcg Injetavel Frasco Ampola | 33,9037 | 286,396 |
| Fingolimode 0,5mg Cpr | 54,29 | 99,7196 |
| Fludrocortisona 0,1mg Cpr | 1,19 | 1,3441 |
| Fluvastatina 40mg Cpr | 3,841 | 3,841 |
| Formoterol 12mcg + Budesonida 400mcg Po Inalante Frasco | 0,6416 | 0,8653 |
| Formoterol 12mcg Cpr Inalante | 0,4297 | 0,7647 |
| Formoterol 6 mcg + Budesonida 200 mcg Cpr Inalante | 0,5354 | 0,5919 |
| Gabapentina 300mg Cpr | 0,2046 | 1,025 |
| Gabapentina 400mg Cpr | 1,62 | 1,31 |
| Galantamina 16mg Cpr | 7,08 | 3,7947 |
| Galantamina 24mg Cpr | 7,58 | 4,575 |
| Galantamina 8mg Cpr | 6,13 | 2,5971 |
| Genfibrozila 600mg Cpr | 1,3139 | 1,6004 |
| Genfibrozila 900mg Cpr | 1,4453 | 2,3033 |
| Glatiramer 20mg Injetavel Frasco Ampola | 56,35 | 110,9139 |
| Golimumabe 50mg Injetavel Seringa Preenchida | 1358,9875 | 2403,42 |
| Gosserrelina 10,80mg Injetavel Seringa Preenchida | 865,21 | 1162,76 |
| Gosserrelina 3,60mg Injetavel Seringa Preenchida | 322,75 | 454,13 |
| Hidroxicloroquina 400mg Cpr | 1,0616 | 1,105 |
| Hidroxido Aluminio 230mg Cpr | 0,1427 | 0,2158 |
| Hidroxido de alumínio 61,5mg/ml Suspensao Oral | 2,89 | 5,67 |
| Hidroxiureia 500mg Cpr | 0,96 | 1,2243 |
| Imiglucerase 400U Frasco Ampola | 1633,39 | 3193,93 |
| Imunoglobulina Anti Hepatite B 100UI Frasco | 98,56 | 105,19 |
| Imunoglobulina Anti Hepatite B 600UI Frasco | 571,5367 | 631,16 |
| Imunoglobulina Humana 0,5g Frasco | 68,56 | 7,416 |
| Imunoglobulina Humana 1g Frasco | 5,0845 | 7,6285 |
| Imunoglobulina Humana 2,5g Frasco | 6,33 | 7,4212 |
| Imunoglobulina Humana 5g Frasco | 5,5678 | 7,0401 |
| Infliximabe 10mg/mL Frasco Ampola | 88,213 | 133,116 |
| Isotretinoina 10mg Cpr | 1,1661 | 1,2533 |
| Isotretinoina 20mg Cpr | 1,3482 | 2,313 |
| Lamivudina 10mg/mL Solucao Oral | 0,3735 | 0,5353 |
| Lamivudina 150mg Cpr | 0,55 | 0,9587 |
| Lamotrigina 100mg Cpr | 0,2364 | 1,5003 |
| Lamotrigina 25mg Cpr | 0,5 | 0,4533 |
| Lamotrigina 50mg Cpr | 0,3274 | 0,7483 |
| Lanreotida 120mg Seringa Preenchida | 1513,36 | 1942,18 |
| Lanreotida 60mg Seringa Preenchida | 1408,22 | 1807,26 |
| Lanreotida 90mg Seringa Preenchida | 1513,36 | 1942,18 |
| Latanoprosta 0,05mg/mL Solucao Oftalmica | 36,45 | 20,5175 |
| Leflunomida 20mg Cpr | 3,3499 | 5,0396 |
| Leuprorrelina 11,25mg Frasco | 890,94 | 1164,34 |
| Leuprorrelina 3,75mg Frasco | 127,32 | 323,74 |
| Lovastatina 10mg Cpr | 0,89 | 0,8303 |
| Lovastatina 20mg Cpr | 1,00 | 1,2623 |
| Lovastatina 40mg Cpr | 3,31 | 2,296 |
| Mesalazina 1000mg Cpr | 7,8657 | 8,184 |
| Mesalazina 250mg Cpr | 0,4679 | 1,733 |
| Mesalazina 400mg Cpr | 0,2509 | 1,3557 |
| Mesalazina 500mg Cpr | 2,6197 | 3,432 |
| Mesalazina 800mg Cpr | 0,4453 | 1,6817 |
| Metadona 10mg Cpr | 0,5994 | 0,767 |
| Metadona 10mg/ml Solucao Injetavel | 2,13 | 2,6424 |
| Metadona 5mg Cpr | 0,3199 | 0,4035 |
| Metotrexato 25 mg/mL Frasco Ampola 2mL | 5,5154 | 2,881 |
| Metotrexato 2,5 mg Cpr | 0,4938 | 0,5317 |
| Micofenolato de Mofetila 500mg Cpr | 0,4539 | 6,708 |
| Micofenolato de Sodio 180mg Cpr | 5,746 | 2,7362 |
| Micofenolato de Sodio 360mg Cpr | 8,1606 | 6,0175 |
| Miglustate 100mg Cpr | 129,17 | 159,8917 |
| Morfina 10mg Cpr | 0,2815 | 0,3169 |
| Morfina 10 mg/mL Ampola 1mL | 1,7169 | 1,3705 |
| Morfina 10 mg/mL - Solução Oral Frasco 60mL | 0,2481 | 0,3118 |
| Morfina 30mg Cpr | 0,7789 | 0,8514 |
| Morfina LC 100mg Cpr | 1,71 | 2,206 |
| Morfina LC 30mg Cpr | 0,9608 | 1,241 |
| Morfina LC 60mg Cpr | 1,3741 | 1,77 |
| Naproxeno 250mg Cpr | 0,3733 | 0,3117 |
| Naproxeno 500mg Cpr | 0,6827 | 0,582 |
| Natalizumabe 300mg Frasco Ampola | 2962,0099 | 3359,42 |
| Octreotida 0,1mg/mL Ampola | 11,9 | 28,91 |
| Octreotida Lar 10mg Frasco Ampola | 1831,64 | 2454,03 |
| Octreotida Lar 20mg Frasco Ampola | 2793,12 | 3775,15 |
| Octreotida Lar 30mg Frasco Ampola | 3773,59 | 5096,43 |
| Olanzapina 10mg Cpr | 3,0163 | 6,743 |
| Olanzapina 5mg Cpr | 1,9427 | 3,405 |
| Ombistavir + Veruprevir + Ritonavir 12,5mg + 75mg + 50mg Cpr | 146,03 | 146,03 |
| Pamidronato dissódico 30mg Solucao Injetavel | 74,46 | 191,465 |
| Pamidronato dissódico 60mg Solução Injetavel | 113,331 | 380,71 |
| Pancreatina 10000UI Cpr | 0,72 | 0,4804 |
| Pancreatina 25000UI Cpr | 1,47 | 1,201 |
| Paricalcitol 5mcg/ml solucao injetavel | 25,75 | 26,75 |
| Penicilamina 250mg Cpr | 1,44 | 1,942 |
| Pilocarpina 20mg/mL Solucao Oftalmica Frasco 10mL | 1,53164 | 0,8 |
| Piridostigmina 60mg Cpr | 0,2431 | 0,3038 |
| Pramipexol 0,125mg Cpr | 0,2205 | 0,3163 |
| Pramipexol 0,25mg Cpr | 0,491 | 0,634 |
| Pramipexol 1mg Cpr | 1,4546 | 2,497 |
| Pravastatina 10mg Cpr | 0,584 | 0,472 |
| Pravastatina 20mg Cpr | 0,7433 | 0,6777 |
| Pravastatina 40mg Cpr | 1,4674 | 1,2943 |
| Primidona 100mg Cpr | 0,5345 | 0,2982 |
| Primidona 250mg Cpr | 0,2162 | 0,7045 |
| Quetiapina 100mg Cpr | 1,5579 | 3,6587 |
| Quetiapina 200mg Cpr | 9,6812 | 6,5157 |
| Quetiapina 25mg Cpr | 0,6588 | 1,0193 |
| Quetiapina 300mg Cpr | 11,34 | 12,0277 |
| Raloxifeno 60mg Cpr | 3,4159 | 2,7538 |
| Ribavirina 250mg Cpr | 4,7517 | 0,4059 |
| Riluzol 50mg Cpr | 9,55 | 13,3059 |
| Risedronato 35mg Cpr | 7,1965 | 6,4283 |
| Risperidona 1mg Cpr | 0,03 | 0,6225 |
| Risperidona 1mg/ml Solucao Oral | 12,77 | 12,77 |
| Risperidona 2mg Cpr | 0,05 | 0,6225 |
| Risperidona 3mg Cpr | 0,06 | 0,798 |
| Rituximabe 500mg Frasco Ampola | 83,6908 | 103,543 |
| Rivastigmina 18mg Adesivo Transdermico | 4,07995 | 4,07995 |
| Rivastigmina 2mg/mL Solucao Oral | 1,3008 | 1,6654 |
| Rivastigmina 4,5mg Cpr | 2,4615 | 3,2143 |
| Rivastigmina 6mg Cpr | 2,5122 | 3,2804 |
| Rivastigmina 9mg Adesivo Transdermico | 3,5 | 3,5 |
| Hidroxido Ferrico 100mg Frasco | 3,57 | 1,16 |
| Salmeterol 50 mcg - Aerossol Frasco | 0,7248 | 0,7248 |
| Salmeterol 50 mcg - pó para inalação Frasco | 0,7248 | 0,8638 |
| Selegilina 10mg Cpr | 1,19 | 1,19 |
| Selegilina 5mg Cpr | 0,42 | 0,596 |
| Sevelamer 800mg Cpr | 1,1417 | 2,0776 |
| Sildenafila 20mg Cpr | 5,88 | 7,6887 |
| Sildenafila 25mg Cpr | 4,85 | 7,16 |
| Sildenafila 50mg Cpr | 5,32 | 4,0776 |
| Simeprevir 150mg Cpr | 82,0257 | 831,6757 |
| Sirolimo 1mg Cpr | 20,7757 | 19,8628 |
| Sirolimo 2mg Cpr | 41,8925 | 39,7257 |
| Sofosbuvir 400mg Cpr | 158,3745 | 1515,0271 |
| Somatropina 12UI Frasco Ampola | 110,76 | 206,3333 |
| Somatropina 4UI Frasco Ampola | 11,89 | 212,74 |
| Sulfassalazina 500mg Cpr | 0,6332 | 0,7368 |
| Tacrolimo 1mg Cpr | 2,1016 | 3,3427 |
| Tacrolimo 5mg Cpr | 11,5 | 13,1672 |
| Tenofovir 300mg Cpr | 3,6209 | 16,3275 |
| Tocilizumabe 20mg/mL Frasco Ampola | 175,4 | 101,688 |
| Tolcapona 100mg Cpr | 2,5 | 3,462 |
| Topiramato 100mg Cpr | 0,1943 | 1,2758 |
| Topiramato 25mg Cpr | 0,1919 | 0,3157 |
| Topiramato 50mg Cpr | 0,1252 | 0,6313 |
| Toxina Botulinica Tipo A 100U Frasco Ampola | 242,8743 | 747,3696 |
| Toxina Botulinica Tipo A 500U Frasco Ampola | 884,13 | 1131,81 |
| Travoprosta 0,04mg/mL Solucao Oftalmica | 26,57 | 15,44 |
| Triexifenidil 5mg Cpr | 0,1948 | 0,2027 |
| Triptorellina 11,25mg Po Suspensao Injetavel | 1002,98 | 1346,24 |
| Triptorellina 3,75mg Po Suspensao Injetavel | 298,56 | 382,2 |
| Vigabatrina 500mg Cpr | 1,8934 | 2,4698 |
| Ziprasidona 40mg Cpr | 4,47 | 2,756 |
| Ziprasidona 80mg Cpr | 7,56 | 4,068 |
